# A Case Report and Literature Review of Cardiac Amyloidosis: The Great Pretender Masquerading As Acute Coronary Syndrome

**DOI:** 10.7759/cureus.81567

**Published:** 2025-04-01

**Authors:** Selma Siagh, Hind Hibatouallah, Malak Amrani, Mohamed Cherti

**Affiliations:** 1 Department of Cardiology B, Ibn Sina Hospital, Mohammed V University, Rabat, MAR

**Keywords:** acute coronary syndrome (acs), anginal chest pain, infiltrative disease, microvascular angina, non-st segment elevation myocardial infarction (nstemi), transthyretin (attr) cardiac amyloidosis

## Abstract

Cardiac amyloidosis is a rare condition characterized by the accumulation of misfolded proteins in the heart. It is often associated with heart failure with preserved ejection fraction, restrictive cardiomyopathy, aortic stenosis, conduction disorders, and arrhythmias, particularly atrial fibrillation. We present the case of a 71-year-old male patient admitted to the cardiology department with crescendo angina, Q waves on the electrocardiogram, and elevated troponin levels. The initial working diagnosis was late-presenting ST-elevation myocardial infarction. However, coronary angiography revealed no significant coronary artery disease, and further diagnostic workup led to the diagnosis of wild-type transthyretin amyloidosis. This case illustrates an atypical presentation of cardiac amyloidosis, masquerading as acute coronary syndrome. It highlights the importance of recognizing key red flags that should raise suspicion for this diagnosis and guide further investigation. Additionally, we review the current literature to explore the pathophysiological mechanisms underlying chest pain in cardiac amyloidosis, offering insights into this often underdiagnosed condition.

## Introduction

Amyloidosis is an infiltrative disease in which misfolded proteins aggregate and form amyloid fibrils that can deposit in various organs, including the heart. The presence and severity of cardiac involvement depend on the type of amyloidosis. More than 98% of cases are attributed to either light chain amyloidosis (AL) or transthyretin amyloidosis (ATTR) [1,2].

Cardiac amyloidosis remains a relatively rare condition, with an estimated prevalence of 1% in the general population [3]. However, it is more frequent in elderly populations, especially in cases of ATTR amyloidosis [4,5]. The most common cardiac manifestations include heart failure with preserved ejection fraction (HFpEF), restrictive cardiomyopathy, aortic stenosis, conduction disorders, and arrhythmias, especially atrial fibrillation (AF) [1-3]. Patients with cardiac amyloidosis may rarely present with chest pain, potentially leading to a misdiagnosis of acute or chronic coronary syndrome. Herein, we describe a case of cardiac amyloidosis mimicking late-presenting ST-elevation myocardial infarction (STEMI) and discuss similar cases in the literature. Our goal is to enhance understanding of the pathophysiology of chest pain in cardiac amyloidosis and to highlight key clinical, electrocardiographic, and echocardiographic features that should raise suspicion for this often-overlooked diagnosis.

## Case presentation

**Figure 1:**
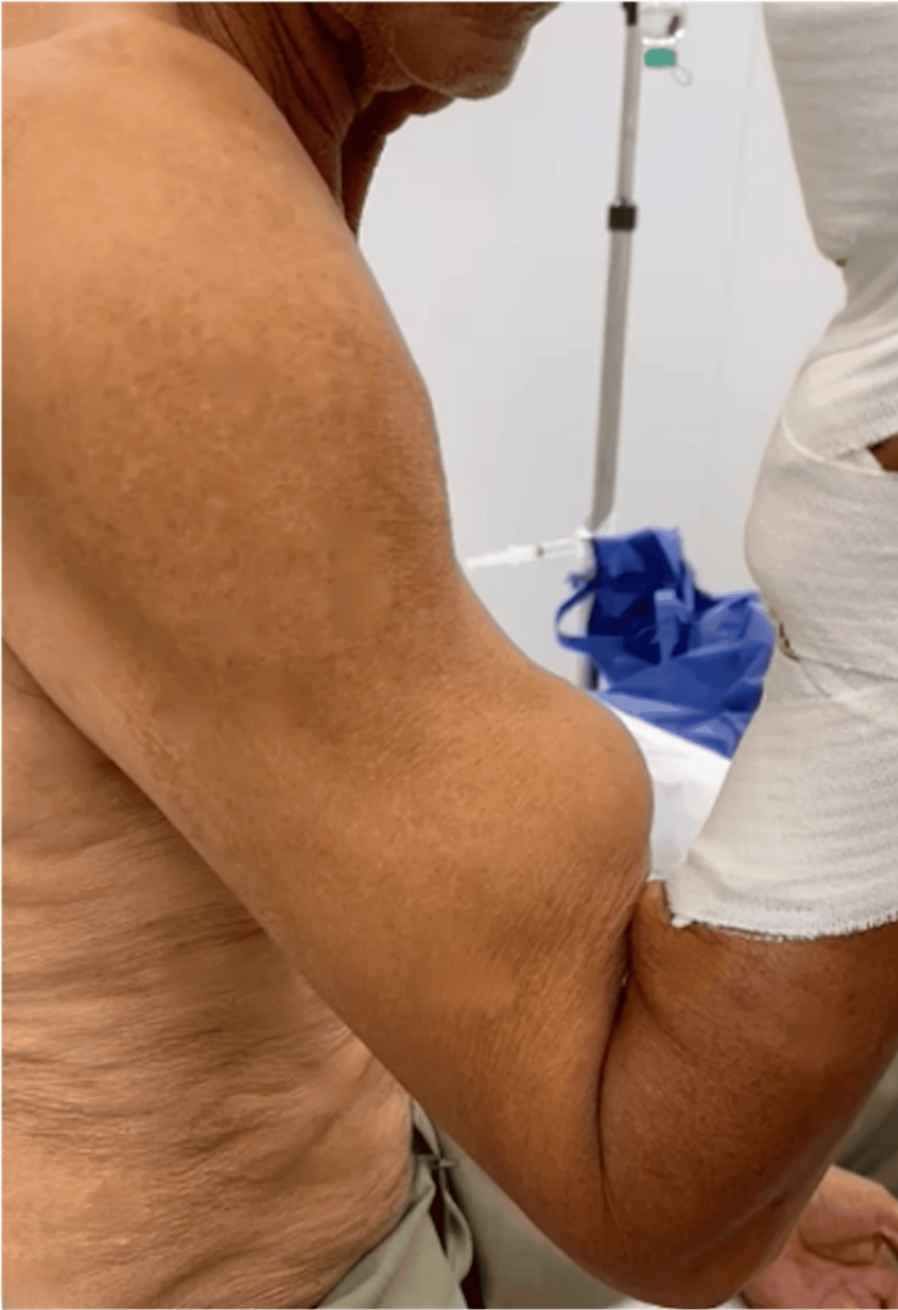
Popeye’s sign (clinical manifestation of biceps tendon rupture)

A 71-year-old male patient was admitted to the cardiology department for exertional angina. He was a chronic smoker, had no history of diabetes, hypertension, or dyslipidemia, and had no other relevant medical history. The patient started complaining of exertional chest pain one year prior, which had worsened a week earlier. Notably, he did not report prolonged chest pain at rest. Upon admission, the patient had a blood pressure of 113/53 mmHg and a heart rate of 58 bpm. The physical examination was unremarkable, except for a rupture of the right biceps tendon as evidenced by Popeye's sign (Figure 1). It should be noted that trauma was ruled out as the underlying cause upon patient questioning.

**Figure 2:**
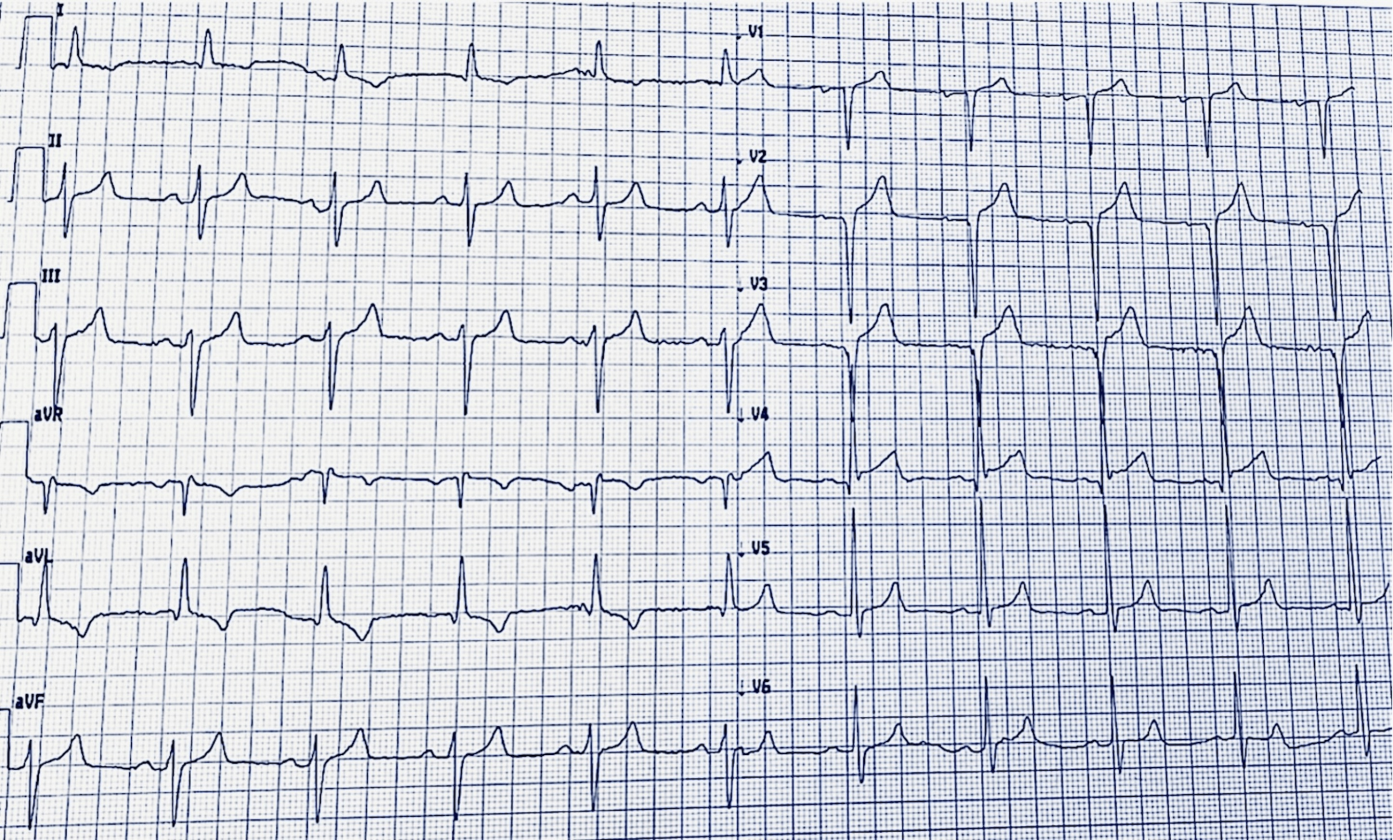
Twelve-lead ECG performed upon admission showing ST-segment elevation in leads V2 to V4, QS complexes in leads V1 to V3, Q waves in lead V4, and negative T waves in the high lateral leads ECG: electrocardiogram

The electrocardiogram (ECG) showed normal sinus rhythm at 62 bpm. There was ST-segment elevation in leads V2 to V4 of 2, 2.5, and 2 mm, respectively. QS complexes were observed in leads V1 to V3, and Q waves were present in lead V4. Additionally, negative T waves were noted in the high lateral leads. There was also a slight left axis deviation and no evidence of left ventricular hypertrophy (Figure 2). Subsequent ECGs performed in the hours and days following admission showed no changes, notably no T wave inversion in leads V2 to V4.

**Figure 3:**
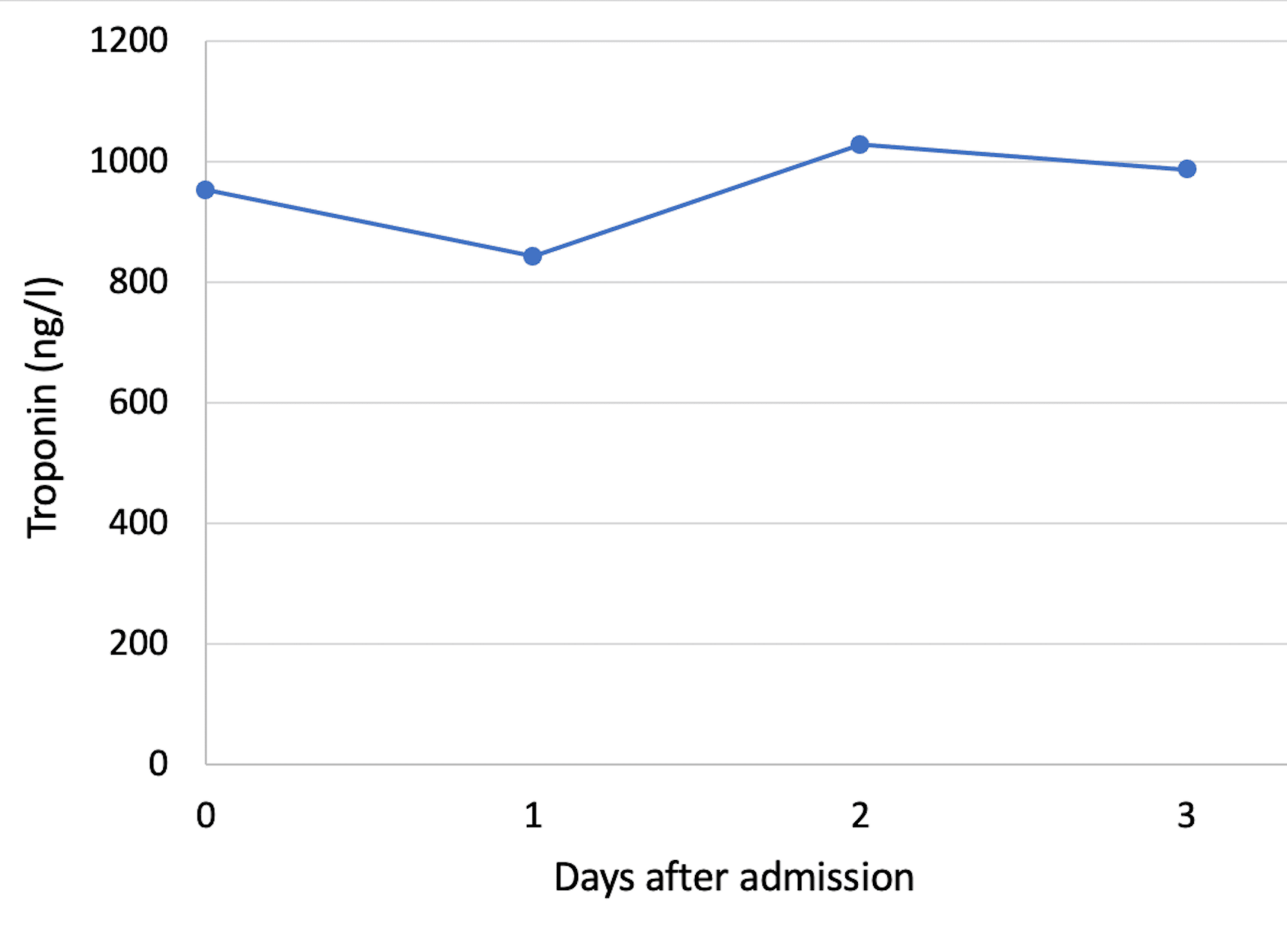
Graph illustrating the variation in troponin levels over time

Initial laboratory testing revealed anemia with hemoglobin at 10.3 g/dl (normal range (NR): 13-16.5), impaired renal function with urea at 0.84 g/l (NR: 0.15-0.55) and creatinine at 19.4 mg/l (NR: 7.2-12.5) as well as elevated C-reactive protein at 84 mg/l (NR: <6). Multiple high-sensitivity cardiac troponin I (hs-cTnI) measurements were performed (Table 1). They revealed persistently elevated levels, ranging between 21.6 and 26.4 times the upper limit with a maximum variation of 18%, and notably with no significant rise or fall (Figure 3). The rest of the biological workup was unremarkable.

**Table 1:** Troponin levels over the days following admission * normal value: <39 ng/l, hs-cTnI: high-sensitivity cardiac troponin I

Dosage timing after admission (in days)	Hs-cTnI (ng/l)	Fold increase over the normal value*
0	953	24.4
1	843	21.6
2	1028	26.4
3	987	25.3

**Figure 4:**
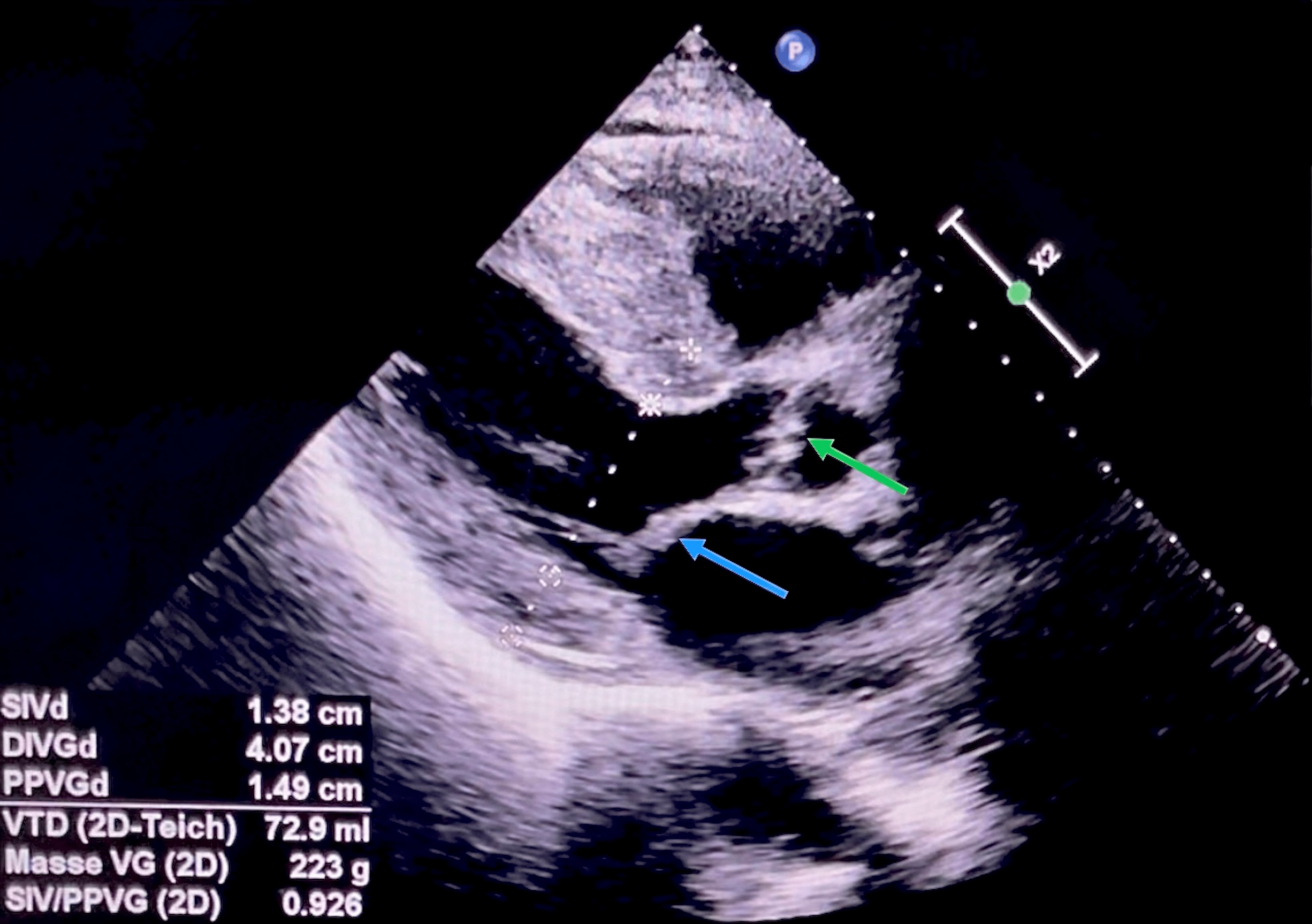
Parasternal view on transthoracic echocardiogram showing concentric LVH, thickened mitral (blue arrow) and aortic (green arrow) valves, and sparkling granular appearance of the myocardium SIVd: interventricular septum thickness at end-diastole (IVSd) = 13.8 mm (normal range (NR): 6-10), DIVGd: left ventricular internal dimension at end-diastole (LVIDd) = 40.7 mm (NR for men: 42-59), PPVG: posterior-wall thickness at end-diastole (PWTd) = 14.9 mm (NR: 6-10), Masse VG: left ventricular mass = 223 g (NR for men: 96-200), corresponding to 132 g/m^2^ (NR for men: 50-115)

**Figure 5:**
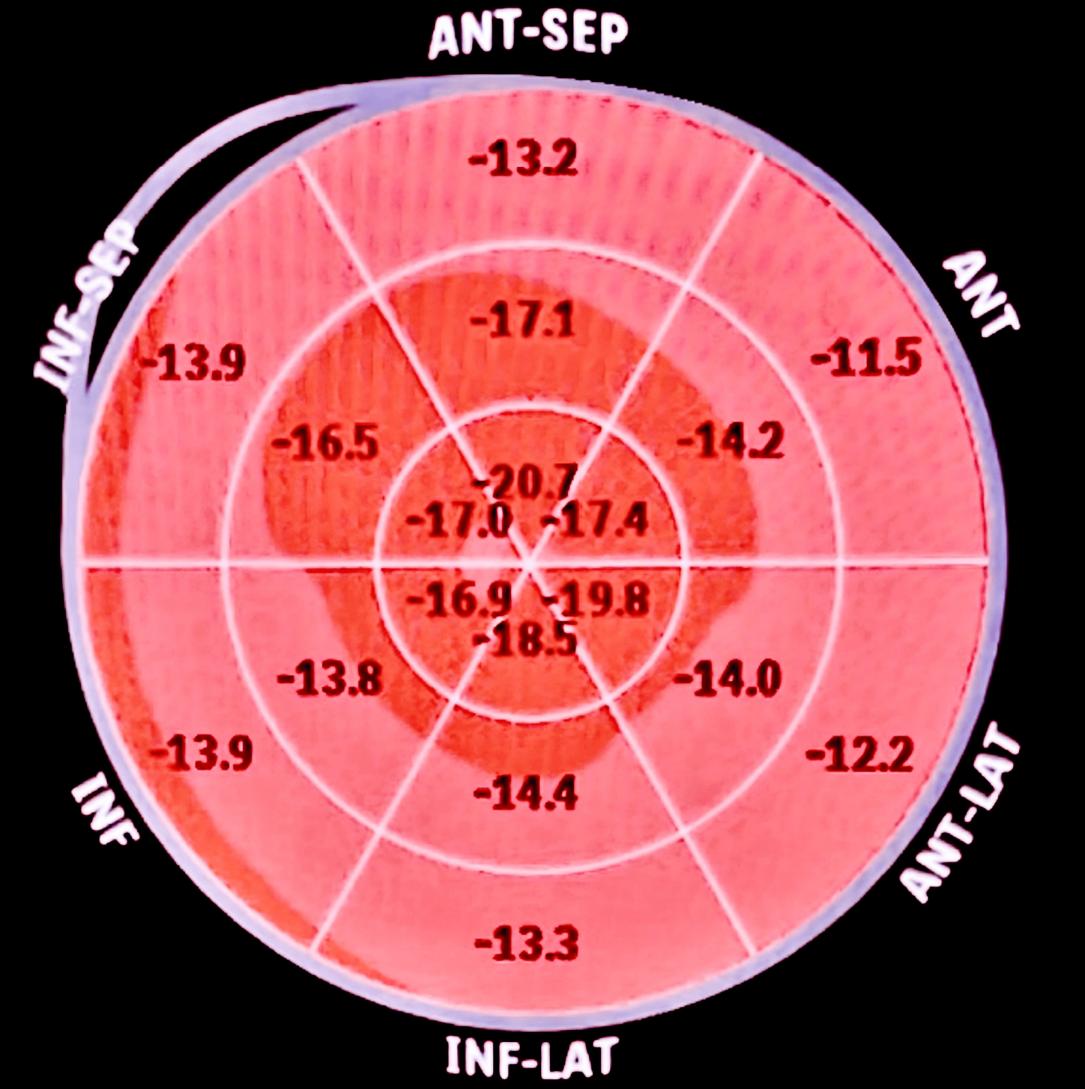
Echocardiographic GLS showing apical sparing pattern GLS: global longitudinal strain, ANT-SEP: anterior septum, ANT: anterior, ANT-LAT: anterolateral, INF-LAT: inferolateral, INF: inferior, INF-SEP: inferior septum

The transthoracic echocardiogram showed significant biventricular hypertrophy, no wall motion abnormalities, preserved ejection fraction, and elevated filling pressures (Video 1, Figure 4). A strain study was performed and showed slightly reduced global longitudinal strain (GLS) at -15.3 (NR: -15.9 to -22.1 %) with an apical sparing pattern (Figure 5).

**Video 1:** Apical four-chamber view on transthoracic echocardiogram showing biventricular hypertrophy with sparkling granular appearance of the myocardium, preserved left ventricular ejection fraction, and no wall motion abnormalities

Given the clinical presentation, which included cardiovascular risk factors and typical chest pain, a coronary angiography was performed to rule out an acute ischemic etiology. It revealed no significant coronary stenosis. With the acute ischemic cause excluded, cardiac amyloidosis was the most likely diagnosis. A bisphosphonate scintigraphy was subsequently performed, revealing grade 2 cardiac uptake. Serum free-light chain quantification and serum and urine immunofixation were negative, and genetic testing for transthyretin mutations was performed. Based on these findings, the diagnosis of wild-type transthyretin cardiac amyloidosis (ATTRwt) was confirmed, and the patient was started on tafamidis. During subsequent follow-up consultations, the most recent of which took place nine months after discharge, the patient reported a progressive reduction in exertional chest pain. Additionally, follow-up echocardiography revealed persistent biventricular hypertrophy and thickened valves, with a preserved ejection fraction.

## Discussion

Amyloidosis is an infiltrative disease in which misfolded proteins aggregate and form amyloid fibrils that can deposit in joints, soft tissue, and various organs, including the heart, kidneys, liver, gastrointestinal tract, and lungs, leading to progressive organ failure. A pathognomonic characteristic is that they display green birefringence under polarized light when stained with Congo red dye. While more than thirty proteins can form amyloid fibrils, 98% of patients with cardiac amyloidosis are affected by either AL or ATTR amyloidosis. The accumulation of immunoglobulin light chains causes the first. The latter, which is the type in the case we presented, results from the accumulation of transthyretin, a tetrameric protein mainly produced in the liver that transports thyroid hormones and retinol. With aging or a destabilizing mutation, the tetramer dissociates into monomers or oligomers that misfold and aggregate into amyloid fibrils. Thus, ATTR amyloidosis can be classified into two subtypes: acquired and hereditary. Acquired ATTR amyloidosis, previously known as senile amyloidosis, is referred to as ATTRwt. Hereditary ATTR amyloidosis is named variant ATTR (ATTRv) [2,6].

In cardiac amyloidosis, amyloid deposits affect the endocardium, myocardium, and pericardium, leading to ventricular hypertrophy, diastolic dysfunction, and, eventually, heart failure. Initially, this presents as HFpEF, which can progress to heart failure with mildly reduced ejection fraction (HFmrEF) or heart failure with reduced ejection fraction (HFrEF). Additionally, amyloid infiltration can cause aortic stenosis and conduction disorders, as well as arrhythmias, particularly AF [2,6]. A systematic review and meta-analysis on the epidemiology of cardiac amyloidosis, conducted by Aimo et al., showed a significant prevalence of cardiac amyloidosis in these conditions: 12% in HFpEF, 10% in HFrEF and HFmrEF, 8% in aortic stenosis, and 2% in conduction disorders. Nevertheless, it remains a rare condition in the general population, with a prevalence of 1% as demonstrated by examining bone scintigraphy results performed for other conditions [3]. Thus, due to its non-specific presentation on the one hand and its rarity overall on the other hand, cardiac amyloidosis is frequently overlooked or misdiagnosed.

Cardiac amyloidosis, particularly ATTR amyloidosis, is especially prevalent in elderly populations. In fact, autopsy data revealed amyloid deposits in 32% of HFpEF patients aged over 75, compared to only 8% of those under 75 [4]. Moreover, Tanskanen et al. showed that transthyretin amyloid deposits were present in the myocardium of 25% of autopsied hearts from unselected adults over the age of 80 [5]. These data suggest that ATTR cardiac amyloidosis is most likely highly underdiagnosed, particularly in elderly populations. It is therefore a diagnosis to remember when managing care for this specific population.

In our case, the clinical presentation was unusual, dominated by chest pain that mimicked acute coronary syndrome. The prevalence of chest pain in patients with cardiac amyloidosis remains unclear. A study from de Michieli et al. indicates that approximately 38% of patients with cardiac amyloidosis experience chest pain [7].

To further elucidate the characteristics of patients with cardiac amyloidosis presenting with chest pain, we conducted a literature review of all cases in the English literature of confirmed cardiac amyloidosis in which the primary complaint was chest pain (acute or chronic). In all the reported cases, the chest pain was attributed to cardiac amyloidosis rather than to an alternative etiology such as coronary atherosclerosis. Our literature search was performed using the PubMed database for articles published up to February 2025, with keywords including cardiac amyloidosis, chest pain, angina pectoris, microvascular angina, chest discomfort, myocardial infarction, and acute coronary syndrome. In addition, we manually searched the reference lists of each included study and scanned relevant papers in PubMed. We selected case reports and patients from case series that met our inclusion criteria. Including our case, the clinical data of 52 patients with cardiac amyloidosis presenting with chest pain [8-38] are summarized in Table 2.

**Table 2:** Published cases (English language) of cardiac amyloidosis presenting with chest pain since 1968 **: cases from case series, ‡: patients with known coronary artery disease, ↗: elevated, +: present, +/-: mild, 0: none, AF: atrial fibrillation, AP: atherosclerotic plaques, AV: atrioventricular, CA: coronary arteries, CHF: congestive heart failure, CK: creatinine kinase, CV: coronary vasospasm, CVD: cardiovascular disease, ECG: electrocardiogram, LBBB: left bundle branch block, LVH: left ventricular hypertrophy, LQRSV: low QRS voltage, MI: myocardial infarction, NS: not specified, R-: small R waves, RBBB: right bundle branch block, STE: ST-segment elevation, STD: ST-segment depression, T-: T wave inversion, VF: ventricular fibrillation, VT: ventricular tachycardia, YO: years old

Study	Age and sex	Modifiable CVD risk factors	Type of amyloidosis	Clinical presentation on admission	ECG	Cardiac markers (troponin and/or CK)	Coronary angiography	Cardiac amyloid deposits (at autopsy, in explanted heart, or cardiac biopsy)	Evolution (at time of publication)
Brandt et al. (1968) [8]	NS	NS	NS	Angina pectoris	NS	NS	AP: NS; testing for CV: NS	Myocardium: +; endocardium: NS; interstitium: NS; epicardial CA: 0; intramural CA: +	Died
	NS	NS	NS	Acute chest pain	Pattern similar to acute inferior MI	NS	AP: NS; testing for CV: NS	Myocardium: +; endocardium: NS; interstitium: NS; epicardial CA: 0; intramural CA: +	Died
Barth et al. (1970) [9]	53 YO, female	NS	AL	Angina pectoris + CHF	NS	NS	AP: 0; testing for CV: NS	Myocardium: +; endocardium: +; interstitium: NS; epicardial CA: +; intramural CA: +	Died
Buja et al. (1970) [10]**	31 YO, male	NS	NS	Angina pectoris + CHF	LQRSV, pattern similar to old MI	NS	AP: +/-; testing for CV: NS	Myocardium: NS; endocardium: NS; interstitium: NS; epicardial CA: 0; Intramural CA: +	Died (heart failure)
	53 YO, female	NS	NS	Angina pectoris + CHF	LQRSV	NS	AP: 0; testing for CV: NS	Myocardium: NS; endocardium: NS; interstitium: NS; epicardial CA: 0; Intramural CA: +	Died (pulmonary embolism)
	67 YO, male	NS	NS	Angina pectoris + CHF	LQRSV, pattern similar to old MI	NS	AP: +/-; testing for CV: NS	Myocardium: NS; endocardium: NS; interstitium: NS; epicardial CA: 0; intramural CA: 0	Died (heart failure)
	89 YO, male	NS	NS	Angina pectoris + CHF	Pattern similar to old MI	NS	AP: +/-; testing for CV: NS	Myocardium: NS; endocardium: NS; interstitium: NS; epicardial CA: 0; intramural CA: +	Died (heart failure)
Smith and Hutchins (1979) [11]**	59 YO, male	NS	AL	Angina pectoris	NS	NS	AP: +/-; testing for CV: NS	Myocardium: +; endocardium: NS; interstitium: NS; epicardial CA: 0; intramural CA: +	Died
	89 YO, male	NS	ATTR	Acute chest pain	NS	NS	AP: +/-; testing for CV: NS	Myocardium: +; endocardium: NS; interstitium: NS; epicardial CA: NS; intramural CA: +	Died
Jennette et al. (1982) [12]	NS	NS	AL	Angina pectoris	NS	NS	AP: 0; testing for CV: NS	Myocardium: NS ; endocardium: NS; interstitium: NS; epicardial CA: 0; intramural CA: +	Died
Saffitz et al. (1983) [13]	58 YO, female	NS	AL	Angina pectoris	First-degree AV block, LQRSV, Q waves in leads II, III, aVF, V5 and V6, R- in the precordial leads	NS	AP: 0; testing for CV: NS	Myocardium: +; endocardium: +; interstitium: 0; epicardial CA: NS; intramural CA: +	Died (sudden death)
Saltissi et al. (1984) [14]	32 YO, male	Chronic smoking	AL	Angina pectoris + CHF	LQRSV in the limb leads, Q waves in leads V1, V2, STD, and T- in the antero-lateral leads	NS	AP: +/-; testing for CV: NS	Myocardium: NS; endocardium: NS; interstitium: NS; epicardial CA: +; intramural CA: NS	Died (sudden death)
Arai et al. (1990) [15]	41 YO, female	NS	NS	Angina pectoris + CHF	NS	NS	AP: 0; testing for CV: NS	Myocardium: NS; endocardium: NS; interstitium: NS; epicardial CA: 0; intramural CA: +	Died (VF)
Narang et al. (1993) [16]	43 YO, female	0	AL	Angina pectoris + CHF	STD in leads II, III, and aVF, QS complexes in the precordial leads	NS	AP: 0; testing for CV: negative	NS	Alive
Ishikawa et al. (1996) [17]	65 YO, male	NS	AL	Angina pectoris + CHF	STD in leads II, III, aVF, and V4 to V6	Normal	AP: 0; testing for CV: NS	Myocardium: 0; endocardium: NS; interstitium: 0; epicardial CA: 0; intramural CA: +	Died
Schäfer et al. (1996) [18] ‡	61 YO, male	Hypertension, dyslipidemia, chronic smoking	AL	Angina pectoris + CHF	Nonspecific ST-segment and T wave changes	NS	AP: 0 (no new stenoses, patent grafts); testing for CV: NS	Myocardium: NS; endocardium: NS; interstitium: 0; epicardial CA: 0; intramural CA: +	Died (VT)
Al Suwaidi et al. (1999) [19]**	63 YO, male	0	AL	Angina pectoris + CHF	NS	NS	AP: 0; testing for CV: NS	Performed in two cases: myocardium: 0; endocardium: +; interstitium: +; epicardial CA: 0; intramural CA: +	Died (heart failure)
	61 YO, male	0	AL	Angina pectoris + CHF	NS	NS	AP: 0; testing for CV: NS		Died (heart failure)
	52 YO, female	0	AL	Acute chest pain + CHF	NS	NS	AP: 0; testing for CV: NS		Died (heart failure)
	65 YO, male	0	AL	Angina pectoris + CHF	NS	NS	AP: 0; testing for CV: NS		Died (heart failure)
	61 YO, male	Dyslipidemia	AL	Angina pectoris + CHF	NS	NS	AP: 0; testing for CV: NS		Died (heart failure)
Mueller et al. (2000) [20]**	64 YO, male	NS	AL	Angina pectoris	Normal	NS	AP: 0; testing for CV: NS	Myocardium: NS; endocardium: NS; interstitium: 0; epicardial CA: +/-; intramural CA: +	Died (heart failure)
	84 YO, male	NS	AL	Angina pectoris + CHF	Left axis deviation, LVH, LBBB	NS	AP: 0; testing for CV: NS	Myocardium: NS; endocardium: NS; interstitium: 0; epicardial CA: +/-; intramural CA: +	Died (acute respiratory distress)
	72 YO, male	NS	AL	Acute chest pain + CHF	Left axis deviation	↗	AP: 0; testing for CV: NS	Myocardium: NS; endocardium: NS; interstitium: +; epicardial CA: +/-; intramural CA: +	Died
	72 YO, male	NS	AL	Angina pectoris + CHF	Left axis deviation, RBBB	↗	AP: 0; testing for CV: NS	Myocardium: NS; endocardium: NS; interstitium: +; epicardial CA: +/-; intramural CA: +	Died
	60 YO, female	NS	AL	Acute chest pain + CHF	AF, LVH	NS	AP: 0; testing for CV: NS	Myocardium: NS; endocardium: NS; interstitium: +; epicardial CA: +/-; intramural CA: +	Died (heart failure)
	64 YO, female	NS	AL	Acute chest pain	Normal	NS	AP: 0; testing for CV: NS	Myocardium: NS; endocardium: NS; interstitium: +; epicardial CA: +/-; intramural CA: +	Died
	48 YO, male	NS	AL	Angina pectoris + CHF	RBBB, lateral wall ischemia	NS	AP: 0; testing for CV: NS	Myocardium: NS; endocardium: NS; interstitium: +; epicardial CA: +/-; intramural CA: +	Died (multisystem failure)
	42 YO, male	NS	AL	Angina pectoris + CHF	Pattern similar to anteroseptal MI	NS	AP: 0; testing for CV: NS	Myocardium: NS; endocardium: NS; interstitium: +; epicardial CA: +/-; intramural CA: +	Alive (after cardiac transplant)
	50 YO, male	NS	AL	Angina pectoris	RBBB	NS	AP: 0; testing for CV: NS	Myocardium: NS; endocardium: NS; interstitium: +; epicardial CA: +/-; intramural CA: +	Died (sudden death)
	68 YO, male	NS	AL	Acute chest pain + CHF	Left axis deviation	↗	AP: 0; testing for CV: NS	Myocardium: NS; endocardium: NS; interstitium: 0; epicardial CA: +/-; intramural CA: +	Died (heart failure)
	57 YO, female	NS	AL	Acute chest pain + CHF	LQRSV, RBBB, pattern similar to anterior infarction	↗	AP: +/-; testing for CV: NS	Myocardium: NS; endocardium: NS; interstitium: +; epicardial CA: +/-; intramural CA: +	Alive (after cardiac transplant)
Ogawa et al. (2001) [21]	69 YO, female	Dyslipidemia	AL	Angina pectoris + CHF	STD in leads II, III, aVF, and V4 to V6	NS	AP: 0; testing for CV: negative	Myocardium: +/-; endocardium: +; interstitium: NS; epicardial CA: 0; intramural CA: +	Died (AV block)
Cantwell et al. (2002) [22]	43 YO, male	Diabetes	AL	Angina pectoris + CHF	LQRSV in the limb leads, STD in the lateral leads, T- in the inferior leads	↗	AP: 0; testing for CV: NS	Myocardium: NS; endocardium: NS; interstitium: NS; epicardial CA: 0; Intramural CA: +	Died (heart failure)
Miani et al. (2002) [23] ‡	65 YO, male	Dyslipidemia, chronic smoking	AL	Angina pectoris	NS	NS	AP: 0 (patent stent); testing for CV: NS	Myocardium: +/-; endocardium: NS; interstitium: NS; epicardial CA: +/-; intramural CA: +	Died (heart failure)
Whitaker et al. (2004) [24]	65 YO, male	Hypertension	AL	Angina pectoris	NS	↗	AP: +/-; testing for CV: NS	Myocardium: +; endocardium: NS; interstitium: NS; epicardial CA: 0; intramural CA: +	Died
Soma et al. (2010) [25]	49 YO, male	NS	AL	Acute chest pain	LQRSV in the limb leads, STE and Q waves in leads II, III, aVF, T- in lead V4 to V6	↗	AP: 0; testing for CV: positive	Myocardium: 0; endocardium: 0; interstitium: 0; epicardial CA: 0; intramural CA: +	Alive
Sohn et al. (2011) [26]	77 YO, male	NS	AL	Angina pectoris + CHF	LQRSV in the limb leads, R- in the precordial leads	↗	AP: 0; testing for CV: positive	Myocardium: NS; endocardium: NS; interstitium: +; epicardial CA: NS; intramural CA: +	Alive (after cardiac transplant)
Edwards et al. (2015) [27]**	63 YO, male	Hypertension	AL	Angina pectoris + CHF	LQRSV throughout, QS complexes in the anterior and inferior leads	NS	AP: NS; testing for CV: NS	Myocardium: NS; endocardium: NS; interstitium: +; epicardial CA : +; intramural CA : +	Died
	46 YO, male	NS	ATTR	Angina pectoris + CHF	NS	Normal	AP: NS; testing for CV: NS	NS	Died
George et al. (2015) [28]	75 YO, male	Dyslipidemia, chronic smoking	AL	Acute chest pain + CHF	STD in leads V4 to V6	↗	AP: +/-; testing for CV: NS	NS	NS
Keller et al. (2016) [29]	65 YO, female	NS	AL	Angina pectoris	NS	NA	AP: 0; testing for CV: NS	Myocardium: NS; endocardium: NS; interstitium: +; epicardial CA: 0; intramural CA: +	NS
Adhikari et al. (2018) [30]	64 YO, male	NS	AL	Angina pectoris + CHF	LQRSV in the limb leads, nonspecific ST-segment and T wave changes	↗	AP: NS; testing for CV: NS	NS	Alive
Ishida et al. (2019) [31]	72 YO, female	NS	AL	Angina pectoris	Normal	NS	AP: 0; testing for CV: NS	Myocardium: NS; endocardium: NS; interstitium: +; epicardial CA: 0; intramural CA: +	Died (heart failure)
Taiwo et al. (2019) [32]	73 YO, female	NS	ATTR	Acute chest pain	LQRSV in the limb leads, LBBB	NS	AP: 0; testing for CV: NS	NS	Alive
Nguyen and Nguyen (2020) [33]	72 YO, male	NS	AL	Acute chest pain	LQRSV in the limb leads, RBBB	↗	AP: 0; testing for CV: NS	NS	Died (sudden death)
Morgado et al. (2021) [34]	64 YO, male	NS	ATTR	Angina pectoris	Complete AV block	NS	AP: +/-; testing for CV: NS	NS	Alive
Tew and Scott (2021) [35]	53 YO, male	NS	AL	Acute chest pain	LVH, widespread T-	↗	AP: 0; testing for CV: NS	NS	Alive (after one year of chemotherapy)
Baptista et al. (2023) [36]	84 YO, male	Dyslipidemia	ATTR	Angina pectoris + CHF	First-degree AV block, LBBB	↗	AP: 0; testing for CV: NS	NS	Alive
Karampela et al. (2024) [37]	75 YO, male	NS	NS	Acute chest pain	NS	↗	AP: 0; testing for CV: NS	NS	Alive
Xie et al. (2024) [38]	54 YO, female	0	NS	Angina pectoris	First-degree AV block, Q waves, and R- in the precordial leads	Normal	AP: 0; testing for CV: NS	NS	Alive
Present case (2025)	71 YO, male	Chronic smoking	ATTR	Angina pectoris + CHF	STE in leads V2 to V4, QS complexes in leads V1 to V3, Q waves in lead V4, and T- in leads DI and VL	↗	AP: 0; testing for CV: NS	NS	Alive

Based on the observations made in these case reports and studies from the literature, we have attempted to elucidate the mechanisms by which cardiac amyloidosis can lead to chest pain. It appears that the pathogenesis of angina in patients with cardiac amyloidosis is not yet fully understood. Several potential mechanisms have been described in the literature, which we have categorized into four groups: luminal narrowing or obstruction due to amyloid deposition affecting the epicardial or intramyocardial arteries, coincident atherosclerotic involvement, reduction of coronary flow reserve (CFR), and other causes such as extrinsic compression of coronary arteries and dysautonomia.

Luminal Narrowing or Obstruction Due to Amyloid Deposition Involving the Epicardial or Intramyocardial Arteries

Amyloid deposition in the intramyocardial coronary arteries, causing luminal narrowing or obstruction, is the most common observation made in the cases we consulted. In fact, we noticed that all the cases reported but three had extensive intramyocardial coronary artery amyloid deposits with no or mild involvement of the epicardial arteries. This has also been extensively described in autopsy studies, such as the one published by Smith et al. The study included 108 cases of cardiac amyloidosis. Five patients (4.6%) had severe occlusive or sub-occlusive amyloid deposits in intramyocardial arteries, associated with recent myocardial ischemic injury. Clinically, four patients had congestive heart failure and one had angina pectoris. In all five patients, the degree of epicardial coronary artery disease was slight in comparison, and atherosclerotic coronary vascular disease was minimal [11]. Similar findings were made by Buja et al. Of 15 patients with cardiac dysfunction secondary to cardiac amyloidosis, six (25%) had narrowing of intramural coronary arteries by amyloid deposits without significant coronary atherosclerosis. Three of the six patients clinically presented with angina pectoris [10].

The vascular amyloid deposition initially occurs in the media and later infiltrates the adventitia and intima, resulting in complete or near-complete luminal obstruction of the intramyocardial arteries. The epicardial arteries are usually spared from significant amyloid deposition. However, subintimal amyloid deposits in the extramural coronary arteries have been reported and can be a cause of luminal narrowing or obstruction. In the study by Buja et al., amyloid deposits were observed in the media of the epicardial arteries in one case (7%) [10]. Additionally, significant amyloid deposits have been observed in the epicardial coronary arteries in three cases we reviewed: Barth et al. [9], Saltissi et al. [14], and Edwards et al. [27]. Notably, in the case described by Saltissi et al., the epicardial coronary deposits were not associated with intramural artery involvement. The authors therefore attributed the patient's angina exclusively to the epicardial coronary amyloid deposition [14].

Moreover, in our literature review, we noted that cases of chest pain secondary to coronary artery obstruction by amyloid deposits were predominantly linked to AL amyloidosis. Indeed, including our own, only six cases of ATTR amyloidosis have been reported in this context since 1968, which makes our case particularly noteworthy. Crotty et al. further supported this observation by demonstrating, through the study of endomyocardial biopsy specimens from 100 patients with cardiac amyloidosis, that vascular involvement was more frequently observed in AL amyloidosis (88%) in comparison with ATTR amyloidosis (26%, p<0.0001) [39].

Coincident Atherosclerotic Involvement

Concomitant atherosclerotic involvement of the epicardial arteries should never be ruled out a priori. In our case, the patient could have had coronary artery disease, given his cardiovascular risk factors, including age, gender, and chronic smoking. Therefore, coronary angiography was warranted to exclude coronary atherosclerosis. Similarly, the contribution of coronary atherosclerosis was also excluded in all the cases we reviewed. One particular case in the literature, reported by Petersen et al., illustrates the complex interplay between these two conditions. A 68-year-old male patient with known coronary artery disease presented with exertional angina and later died from sudden death due to ventricular fibrillation. At autopsy, his epicardial coronary arteries showed severe atherosclerosis, with significant narrowing of the three main vessels (right coronary artery, left anterior descending artery, and circumflex artery). In addition, diffuse amyloid deposition in the intramural coronary arteries led to severe narrowing of the small vessels. The degree of contribution of each factor to the patient's symptoms is difficult to determine, highlighting the intertwined nature of these conditions [40].

Reduction of Coronary Flow Reserve

CFR refers to the capacity of the coronary blood flow to increase in response to elevated myocardial metabolic demands. Under normal conditions, blood flow can increase approximately four to six times to meet these demands. The process is mediated by vasodilation in the arteriolar bed. A reduction in CFR associated with cardiac amyloidosis has been demonstrated in the literature [41,42].

Dorbala et al. studied CFR using vasodilator stress with N-13 ammonia positron emission tomography, comparing two groups: 21 patients with cardiac amyloidosis without epicardial coronary artery disease and a control group of 10 subjects with hypertensive left ventricular hypertrophy (LVH) [41]. Similarly, Clemmensen et al. assessed CFR using adenosine perfusion and Doppler echocardiography in 27 patients with confirmed cardiac amyloidosis and 42 healthy controls [42]. Both studies found that CFR was severely reduced in patients with cardiac amyloidosis compared to control subjects [41,42], even when normalized to left ventricular mass [41].

The reduction in CFR can be due to impaired arteriolar vasodilation properties or a state of baseline hyperemia [41,42]. The main mechanism involved in the case of cardiac amyloidosis is the impairment of coronary vasodilation, as evidenced by a reduced hyperemic flow velocity [42,43]. This alteration in vasodilatory function can be either endothelial-dependent or endothelial-independent. In a study by Al Suwaidi et al., the authors examined both endothelial-independent CFR, assessed by intracoronary injection of adenosine, and endothelial-dependent CFR, assessed by infusion of acetylcholine. Both CFR forms were significantly reduced in all patients [19]. Al Suwaidi et al. and Clemmensen et al. attributed these observations to amyloid infiltration of vascular smooth muscle cells [19,42]. A baseline hyperemic state may also contribute to the reduction in CFR. Indeed, patients with cardiac amyloidosis have been shown to exhibit significantly higher resting coronary flow velocities [42,43]. Paradoxically, however, Dorbala et al. demonstrated that the amyloid group had lower resting myocardial blood flow, suggesting reduced myocardial perfusion at rest [41]. Clemmensen et al. hypothesized that the paradox of high coronary flow velocity but low myocardial perfusion may be indicative of non-perfused amyloid deposits. These deposits, responsible for increased ventricular wall thickness, lead to a relative paucity of blood vessels compared to the overall ventricular mass. Furthermore, the presence of non-perfused amyloid deposits subsequently elevates the metabolic demand on myocytes required to maintain normal cardiac output. Moreover, the elevated resting flow velocity could be directly related to an amyloid-induced reduction in coronary artery compliance [42].

Other Causes

Another mechanism of chest pain in cardiac amyloidosis, which also involves increased ventricular wall thickness, is diastolic dysfunction, particularly impaired relaxation. This impaired relaxation disrupts the normal suction effect, facilitating blood flow into the intramyocardial coronary arteries during diastole. Elevated end-diastolic pressures further reduce blood flow, especially in the subendocardial layers. This combination of factors ultimately results in ischemic chest pain [41].

Extrinsic compression of the microvasculature due to perivascular and interstitial amyloid deposits could also explain chest pain in cardiac amyloidosis [41].

Finally, autonomic dysfunction may contribute to chest pain in patients with cardiac amyloidosis. It is commonly observed in ATTR, often manifesting as impaired baroreflex function and abnormal vascular autonomic regulation, particularly with regard to sympathetic regulation [43]. The impact of autonomic dysfunction on coronary circulation has been well-documented in other neuropathies, notably diabetic neuropathy. Indeed, Di Carli et al. demonstrated that diabetic autonomic neuropathy is associated with an impaired vasodilatory response in coronary resistance vessels during sympathetic stimulation [44]. Although not specifically studied in cardiac amyloidosis, autonomic dysfunction remains a plausible hypothesis. It may play a role in microvascular dysfunction, contributing to the pathogenesis of chest pain in these patients.

**Figure 6:**
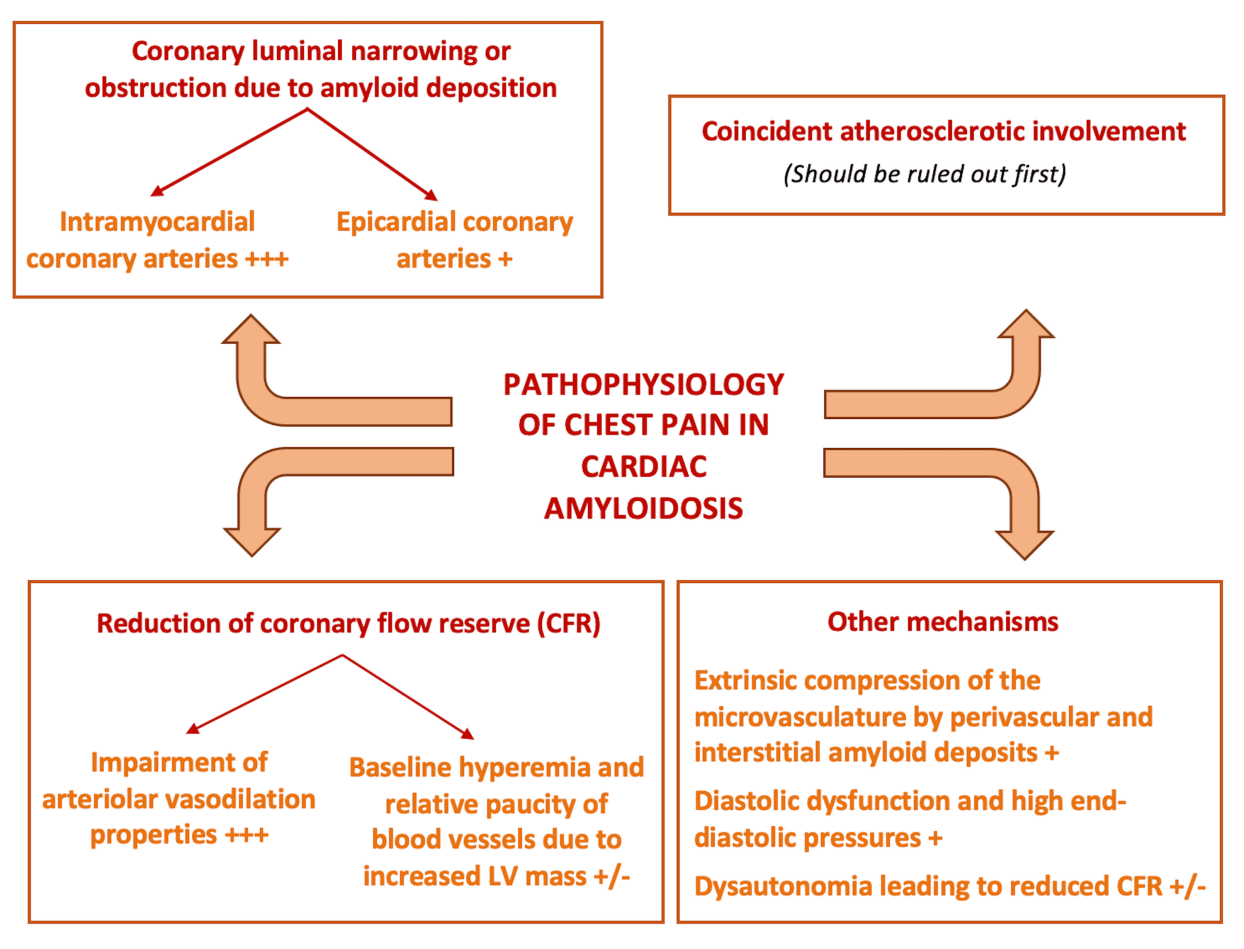
Schematic overview of the mechanisms of chest pain in cardiac amyloidosis (proposed by the authors) +++: important contribution, +: proven contribution, +/-: possible contribution, LV: left ventricular

We have provided a summary of all the mechanisms described above in the form of a diagram, which visually represents the key processes involved (Figure 6).

Cardiac amyloidosis has been described as a “great pretender." It is indeed often misdiagnosed as it can mimic multiple cardiovascular conditions, notably in this case, ischemic heart disease [2]. In fact, in addition to the clinical presentation predominantly characterized by chest pain, which was reported in our case and all the cases reviewed in the literature, some electrical and biological features of cardiac amyloidosis may also suggest an ischemic etiology. The presence of Q waves on the ECG associated with ST-segment elevation initially suggested a STEMI diagnosis. However, upon further investigation, the absence of ST-segment and T wave changes or evolving Q waves over several hours suggested that these were likely pseudo Q waves, which are characteristic of cardiac amyloidosis. Another significant clue was the presence of LVH on echocardiography, despite the lack of electrical signs of LVH [1]. Moreover, the initial elevated troponin levels associated with the ECG pattern suggested an acute ischemic cause. However, the lack of significant changes in troponin values over time pointed towards chronic rather than acute myocardial injury, which is more consistent with cardiac amyloidosis. Additionally, echocardiographic features, including biventricular hypertrophy, a granular myocardial texture, HFpEF, and apical sparing on strain imaging, further supported the diagnosis. It should also be noted that the patient exhibited on clinical examination Popeye’s sign, which indicates biceps tendon rupture. This provided additional evidence for the diagnosis of amyloidosis. All of the parameters we have mentioned are part of the so-called “red flags” summarized in the work of Garcia-Pavia et al. (Table 3) [1]. Identifying these key features is essential to differentiate cardiac amyloidosis from other cardiovascular conditions.

**Table 3:** "Red flags" that should raise suspicion of an ATTR amyloidosis diagnosis when associated with LVH greater than 12 mm ECG: electrocardiogram, ECV: extracellular volume, LGE: late gadolinium enhancement, ATTR: transthyretin amyloidosis, LVH: left ventricular hypertrophy According to Garcia-Pavia et al. (2021) [1]

“Red flags”	Present case
Heart failure in ≥ 65 years old	Present
Aortic stenosis in ≥ 65 years old	Absent
Hypotension or normotensive if previously hypertensive	Absent
Sensory involvement, autonomic dysfunction	Absent
Peripheral polyneuropathy	Absent
Proteinuria	Absent
Skin bruising	Absent
Bilateral carpal tunnel syndrome	Absent
Ruptured biceps tendon	Present
Subendocardial/transmural LGE or increased ECV	Absent
Reduced longitudinal strain with apical sparing	Present
Decreased QRS voltage to mass ratio	Present
Pseudo Q waves on ECG	Present
Atrioventricular conduction disease	Absent
Possible family history	Absent

The case we reported is noteworthy due to the atypical clinical presentation of cardiac amyloidosis, which mimicked acute coronary syndrome. Such a presentation is rarely observed in ATTR amyloidosis, making our case particularly interesting. Moreover, it offers several valuable insights. First and foremost, it underscores the importance of maintaining a broad differential diagnosis. A comprehensive patient evaluation is essential, and one must take a step back to reconsider the initial findings, as several clues in our case pointed towards cardiac amyloidosis from the outset. To achieve this, being familiar with the “red flags” that may raise suspicion for cardiac amyloidosis is crucial. That said, it remains appropriate to rule out atherosclerotic coronary artery disease, as we did in our case. Finally, this case is particularly compelling due to its rich semiological features, showcasing multiple clinical, electrical, and echocardiographic findings that are commonly associated with cardiac amyloidosis.

## Conclusions

Cardiac amyloidosis can be a cause of microvascular angina. In fact, while the typical presentations include HFpEF, restrictive cardiomyopathy, aortic stenosis, conduction disorder, and arrhythmias, it can also present with chest pain, mimicking coronary artery disease. The main mechanisms include amyloid deposition in the intramyocardial coronary arteries and impaired coronary vasodilation due to amyloid infiltration of vascular smooth muscle cells. Other contributing factors may involve extrinsic compression of the coronary arteries and dysautonomia. A key takeaway is the importance of ruling out coronary artery disease while considering cardiac amyloidosis in the differential diagnosis of patients presenting with chest pain, particularly in elderly populations. Finally, this case highlights more broadly the necessity of recognizing the distinctive features of cardiac amyloidosis, as its nonspecific presentation often leads to incorrect diagnoses and diagnostic delays.
